# Editorial for the Special Issue “Advances in *Acanthamoeba*”

**DOI:** 10.3390/microorganisms12050865

**Published:** 2024-04-26

**Authors:** Daniele Corsaro

**Affiliations:** CHLAREAS, 12, rue du Maconnais, 54500 Vandœuvre-lès-Nancy, France; corsaro@gmx.fr

Some free-living amoebae can behave as opportunistic parasites, causing rare but dangerous diseases in humans and animals, primarily amoebic keratitis, with loss of vision, and encephalitis, which is almost always fatal [1]. *Acanthamoeba* is the most widespread and diverse pathogenic amoeba, several species of which are associated with different pathologies. Biomolecular analyses have revealed extreme diversity within *Acanthamoeba*, challenging traditional morphological criteria for describing species while allowing distinct lineages to be more clearly delineated as genotypes [2,3]. Given its impact on human health, *Acanthamoeba* has long been the subject of extensive research. However, several important aspects of its biology still remain to be elucidated. Further studies are needed to better understand its overall physiology, with particular attention to the factors underlying its pathogenicity. On the other hand, improvements in biomolecular tools allow for the rapid identification of strains, monitoring of their distribution in the environment, and recognition of sources of contamination. Advances in genetic and genomic data will allow for an ever finer understanding of the evolutionary history of *Acanthamoeba* and its allies.

The growing interest in *Acanthamoeba* has motivated the publication of this Special Issue of *Microorganisms*, “Advances in *Acanthamoeba*”, which includes articles covering these pressing topics.

*Acanthamoeba* species are defined based on cyst characteristics and are divided into three morphological groups [4]. Such a classification is not always consistent with genetic data and, as a result, many strains have been found to be incorrectly assigned to a given species [3]. Type strains of almost all species, as well as various strains isolated in several studies, have been deposited in culture collections at the American Type Culture Collection (ATCC), Virginia, USA, and at the Culture Collection of Algae and Protozoa (CCAP), Scotland, UK. These serve as standard strains for any investigation into *Acanthamoeba*. An update on the current maintenance status of these collections is presented in this Special Issue [5], listing the strains present in one or both collections and those that have been lost, as well as those for which genetic data are now newly available.

Amoebic cysts allow for the survival of the organism in hostile conditions, such as a lack of nutrients and the presence of toxic substances. Cyst characteristics vary depending on the type of amoeba; those of *Acanthamoeba* have proven to be the most resistant and therefore have been studied particularly extensively. In a paper presented in this Special Issue, a laboratory model of the water column is developed to study the distribution of cysts of three free-living amoebae, *Acanthamoeba*, *Balamuthia*, and *Vermamoeba* [6]. Another paper analyses gene expression during *Acanthamoeba* encystation, showing the upregulation of glutathione S-transferase genes and other closely related proteins [7].

The most widely used approach to identifying *Acanthamoeba* strains is the partial amplification of nuclear SSU rDNA (18S rDNA), which contain the hypervariable stem 29-1 followed by sequence analysis [8]. Once a partial sequence is correctly obtained, genotypes and their variants can often be determined with great precision. Two papers published in this Special Issue report the molecular identification of *Acanthamoeba* strains from two separate countries via partial 18S sequencing. These include new data on the presence of T4D strains in water and sediment from two rivers in the Atacama Desert, Chile [9], as well as an in-depth investigation of the genotypes (six in total) and allelic variants circulating in Italy, both in the environment and in clinical cases [10].

However, partial 18S results in less robust trees; reliable phylogenetic analysis requires complete genetic sequences to identify the different lineages and the main relationships between them. Full-length mitochondrial SSU rDNA produces a nearly identical result [11], often showing consistent co-clustering [3]. In this Special Issue, two papers analyze additional genetic markers extracted from the genomic data of various *Acanthamoeba* strains, showing that their evolutionary branching patterns are consistent with those obtained with nuclear and mitochondrial SSU rDNA. In one study [12], the complete sequences of the large subunit (LSU) rDNA and internal transcribed spacer (ITS) of various genotypes were obtained for the first time. Furthermore, a number of ITS-LSU sequences deposited in GenBank as “uncultivated fungi” were identified to actually be *Acanthamoeba* sequences. Yet another study [13] investigated the variations in genetic structure and amino acid sequences between the genotypes of two major *Acanthamoeba* adhesins, mannose- and laminin-binding proteins. The changes observed in both proteins indicate that they occurred during the diversification of *Acanthamoeba* species.
